# Empagliflozin: a wonder drug for the treatment of SIAD?

**DOI:** 10.3389/fendo.2024.1453159

**Published:** 2024-10-07

**Authors:** Ploutarchos Tzoulis

**Affiliations:** Department of Metabolism & Experimental Therapeutics, Division of Medicine, University College London, London, United Kingdom

**Keywords:** empagliflozin, SGLT 2 inhibitor, hyponatremia, SIAD(H), AVP

## Introduction

The syndrome of inappropriate antidiuresis (SIAD), the commonest cause of euvolemic hyponatremia, is associated with significant morbidity and mortality (1, 2). Despite the poor efficacy of fluid restriction (3–5), second line agents, such as urea (6, 7) and tolvaptan (8–10), are underutilized in clinical practice. This unmet clinical need may be satisfied by empagliflozin, with emerging evidence supporting its use as a promising new treatment option for SIAD (11, 12). Empagliflozin, a potent, selective inhibitor of sodium glucose cotransporter 2 (SGLT2), is an oral glucose-lowering agent widely used for the treatment of type 2 diabetes mellitus (DM) (13–15) (16), especially in patients with established cardiovascular disease (17) or heart failure or chronic kidney disease (CKD) (18) because of its favorable cardiorenal outcomes. In recent years, empagliflozin has gained approval for two new indications, regardless of the presence or absence of DM; firstly, for the treatment of heart failure across the spectrum of ejection fraction (19, 20), and second, as a nephroprotective agent in patients with CKD at risk of progression (21).

## Mechanisms of empagliflozin action in SIAD

SGLT2, a high capacity and low affinity glucose transporter located in the first segment of the proximal convoluted tubule, accounts for the reabsorption of approximately 90% of filtered glucose (22, 23). Therefore, empagliflozin, through SGLT2 inhibition, induces pronounced glycosuria, leading to osmotic diuresis and increased renal electrolyte-free water excretion (24). Of note is that SGLT2 inhibitors may induce estivation-like metabolic patterns, evolutionarily conserved survival strategies enabling physiological adaptation to energy and water shortage, which potentially contribute to observed improvements in cardiac and renal function (25). After the first dose of empagliflozin, natriuresis occurs due to SGLT2 inhibition, where sodium is coupled with glucose reabsorption (26–28), and blockage of sodium-hydrogen exchanger-3 (NHE3) in the proximal tubule (29). However, natriuresis is short-lived and transient, recovering to the baseline a few days later (30), following compensatory increase of sodium reabsorption in the distal tubule and collecting duct (31), increase in plasma renin activity and reduction in natriuretic peptides (26–28). Animal studies showing that empagliflozin reduces expression of Na^+^-K^+^-2Cl^-^ cotransporter and epithelial Na^+^ channels (ENaC) (32) and decreases mRNA and protein levels of aquaporin 2 (32), the main channel for water reabsorption, suggest that empagliflozin may partially contribute to polyuria via its direct effect on sodium and water channels (26, 32).

Given the well-known effect of SGLT2 inhibition on enhancing urinary water excretion in patients with type 2 DM, an artificial SIAD model was created in healthy volunteers via intravenous desmopressin administration and overhydration (33). This double-blind placebo-controlled randomized crossover study, including 14 healthy volunteers, examined the effect of empagliflozin on urine volume and glycosuria for a 6-hour period (33). In this artificial SIAD model, a single dose of empagliflozin 25 mg led to significantly increased urine volume in association with glycosuria, but without a significant difference in total natriuresis, demonstrating a strong aquaretic action of empagliflozin (33).

An interesting observation is that administration of SGLT2 inhibitors in patients with type 2 DM promotes release of arginine vasopressin (AVP) in order to compensate for persistent osmotic diuresis (34). Empagliflozin-induced losses of electrolyte-free water produce water flow from intracellular to extracellular fluid which are detected by osmoreceptors stimulating AVP release (34). Following these findings, a recent study investigated whether empagliflozin improves hyponatremia through affecting apelin and AVP, two neuropeptides which regulate water homeostasis in opposing ways; AVP by promoting antidiuresis, whereas apelin by augmenting diuresis (35–37). In patients with SIAD, empagliflozin had no effect on plasma apelin concentration, but led to a significant increase by 25% of median serum levels of copeptin, an equimolar surrogate marker of AVP (38). These findings suggest that the efficacy of empagliflozin in SIAD is neither mediated through apelin nor blunted by the adaptive stimulation of AVP release (38). The finding that the increase in serum AVP levels does not compromise the efficacy of empagliflozin in SIAD is also supported by the fact that the overall effect of empagliflozin on ENaC is reduced activity (32) contrary to the marked upregulation of ENaC expression in states of chronic exposure to AVP (39).

## Studies of empagliflozin as treatment for SIAD

Two randomized controlled trials (RCTs) have evaluated the efficacy and safety of empagliflozin for treatment of SIAD (11, 12). The first trial, recruiting 88 hospitalized patients with SIAD and serum sodium < 130 mmol/l (median sodium 126 mmol/l) and assigning them to empagliflozin 25 mg or placebo once daily for 4 days in addition to standard fluid restriction less than 1000 ml/day, showed that empagliflozin led to a significantly higher median sodium increase of 10 mmol/l versus 7 mmol/l in the placebo group (P = 0.04) (11). The difference in serum sodium concentration between empagliflozin and placebo arm was already noted after 24 hours (4 versus 3 mmol/l), increased after 48 hours (8 versus 5 mmol/l) and persisted for the whole 4-day period. It is worth mentioning that the control arm of mild fluid restriction showed a median sodium increment of 7 mmol/l, much higher than previously reported (3), suggesting a potentially significant contribution of hyponatremia autocorrection after reversing the cause of SIAD (40). Empagliflozin was well tolerated with no events of hypoglycemia or hypotension. Out of 43 empagliflozin-treated patients, there were two cases (4.6%) of overly rapid hyponatremia correction and 4 cases (9.3%) of transient decrease in renal function (11). Patients in the empagliflozin arm exhibited marked glycosuria, as evidenced by median urinary glucose of 111.2 mmol/l versus 0.3 mmol/l in the placebo arm, and empagliflozin treatment produced a statistically significant increase in urine osmolality by 164 mOsm/kg compared with the placebo group (P < 0.001) (11).The magnitude of serum sodium increase was negatively correlated with baseline serum sodium and osmolality, with more pronounced changes in serum sodium being observed in those with profound hyponatremia (serum sodium < 125 mmol/l) at the outset (11). In total, this study demonstrated that empagliflozin in combination with fluid restriction is an effective treatment option for hospitalized patients with SIAD over a 4-day period.

Following these encouraging findings, a randomized, double-blind, placebo-controlled, crossover trial evaluated the efficacy and safety of empagliflozin compared to placebo combined with limitation of fluid intake to around 1500 ml/day in 14 outpatients with median serum sodium of 131 mmol/l due to chronic SIAD (12). Four-week treatment with empagliflozin resulted in a median serum sodium increase of 4.1 mmol/l compared to placebo (P = 0.004) which was achieved at the end of first week and remained stable until the end of the study period (12). Following four-week empagliflozin administration, median urine osmolality was 601 mOsm/kg, significantly higher than 494 mOsm/kg in placebo group (P < 0.001) (12). Empagliflozin was well tolerated with no events of hypoglycemia, hypotension, overly sodium correction or acute kidney injury. In addition, empagliflozin led to a small improvement in neurocognitive function, especially when normonatremia was restored, but without any significant improvement in gait (12). In total, this study reported that a 4-week treatment with empagliflozin led to a significant, albeit modest, increase in serum sodium levels in outpatients with chronic SIAD (12).

Secondary analysis of both RCTs has shown that empagliflozin-induced correction of hyponatremia in patients with SIAD is associated with a statistically significant increase in Procollagen type 1 N-terminal propeptide (P1NP), a surrogate marker of bone formation, but without a significant effect on C-terminal telopeptide (CTX), a marker of bone resorption (41) (42). In total, bone formation index, the ratio of P1NP to CTX, increases, indicating that empagliflozin-induced increase in serum sodium levels over a short duration of 4 days (41) or 4 weeks (42) has a positive impact on bone metabolism through activation of osteoblast function, while the long-term effect on bone turnover warrants further study (41, 42).

Empagliflozin-induced increase of serum sodium in patients with SIAD cannot be extrapolated to patients without SIAD since analysis of pooled data from several RCTs does not show any changes in serum sodium concentration in empagliflozin-treated patients with type 2 DM and normonatremia (43, 44). A retrospective analysis of hospitalized patients with type 2 DM has shown that long-term therapy with SGLT2 inhibitors does not alter the prevalence of hyponatremia on hospital admission (45). Therefore, SGLT2 inhibitors do not prevent the development of hyponatremia and should not be used as prophylaxis for hyponatremia in at-risk individuals (45). Additionally, data about the effect of empagliflozin on hypervolemic hyponatremia due to heart failure or cirrhosis are lacking.

## Pros and cons of empagliflozin for SIAD

Empagliflozin may represent a promising therapeutic option for SIAD, having the following advantages: (i) rapid onset of action since difference in serum sodium compared to placebo was already noted after 12 hours (11), (ii) efficacy in increasing serum sodium concentration compared to placebo in both hospitalized patients (11) and outpatients with SIAD (12) as well as in combination with both standard fluid restriction < 1000 ml/day (11) and limitation of fluid intake to 1500-1600 ml/day (12), (iii) being well tolerated with no events of hypoglycemia or hypotension in SIAD studies (11, 12), (iv) favorable safety profile in general based on numerous RCTs and post-marketing surveillance in patients with type 2 DM (17) (v) cardiovascular benefits in individuals with type 2 DM and established cardiovascular disease (17, 46), cardioprotective properties in those with heart failure (19) (20) and nephroprotective effects in those with type 2 DM (47) or CKD (21) (48), (vi) widespread availability, clinicians’ familiarity and ease of use in comparison to the alternative drug therapies for SIAD (vii) relatively low cost, being around 1/40th of tolvaptan cost in the UK.

Empagliflozin as a novel drug agent for SIAD carries some disadvantages: (i) modest efficacy, as suggested by a mean placebo-subtracted serum sodium increase of 3 mmol/l after 4 days (11) and 4.1 mmol/l after 4 weeks (12), (ii) limited evidence base supporting its efficacy, based on its use in 43 patients over 4 days (11) and 14 individuals over 4 weeks (12), (iii) lack of data evaluating its efficacy and safety in combination with tight fluid restriction, such as below 1000 or 500 ml/day, (iv) potential risk of excessive hyponatremia correction and renal deterioration (11).

## Unanswered questions and research gaps

The evidence to date, limited to two small RCTs, highlights the need for large-scale, prospective studies, evaluating the long-term durability of response and safety profile. The efficacy and safety of empagliflozin for treatment of SIAD should be assessed across different levels of baseline serum sodium and at different degrees of fluid restriction, while it remains to be tested whether relaxation of fluid restriction is required prior to empagliflozin initiation in order to prevent decrease in renal function, especially in the elderly. Another safety concern to be addressed is whether hospitalized patients treated with empagliflozin for SIAD are at an increased risk for euglycemic diabetic ketoacidosis (DKA), a well-known association in inpatients with type 2 DM treated with SGLT2 inhibitors (49). Future studies should incorporate as secondary end points the association of various baseline parameters, including biochemical variables, cause of SIAD and level of renal function, with the therapeutic potency and safety of empagliflozin in order to identify predictors of response and facilitate personalized decision-making. In addition, head-to-head studies are warranted to directly compare the efficacy and safety of empagiflozin with other drug options for SIAD, such as urea and tolvaptan. Taking into account the similar efficacy and safety profiles of all SGLT2 inhibitors (50), the role of dapagliflozin and canagliflozin as effective therapies for SIAD also warrants evaluation. A prospective, multicentric, randomized, double-blind placebo-controlled trial (the EMPOWER study; NCT04447911) is currently underway, evaluating the effect of empagliflozin not only in in euvolemic, but also in hypervolemic hyponatremia over a 30-day treatment period. The results of this study are eagerly awaited, especially with respect to elucidating the effect of empagliflozin on natremia in patients with heart failure and liver cirrhosis (51). Without generating high-quality data, empagliflozin may remain a promising agent with limited evidence base, used in an ‘‘off-label’’ manner only in selected cases, following the example of urea. For this reason, the scientific community should urge the pharmaceutical industry to undertake large RCTs, enabling application to regulatory authorities for this new indication.

## Current and future role of empagliflozin

Could empagliflozin be another example of successful drug repurposing? Evidence to date suggests that, at present, empagliflozin combined with fluid restriction should be primarily used in outpatients with mild to moderate, chronic, paucisymptomatic hyponatremia due to SIAD, leading to a significant, but modest, sodium increase and possibly allowing relaxation of fluid restriction. However, the lack of long-term data except for a single case report (52) should limit its use to 4 weeks. The current role of empagliflozin is limited, if any, and merits further investigation in the following three scenarios:

- In hospitalized patients since current guidelines recommend withholding SGLT2 inhibitors during admission for acute serious medical illness or major surgical procedures in view of the increased risk for DKA (53) (54), unless it is proven that this excess risk does not apply to patients without DM.
- In profound hyponatremia with serum sodium below 120 mmol/l, since therapeutic options with greater potency, such as hypertonic saline or tolvaptan, would be required to provide the desired increment of sodium rise.
- In hypervolemic hyponatremia due to heart failure or liver cirrhosis since all data on empagliflozin-related sodium increase are limited to SIAD. In the setting of acute heart failure, the data about empagliflozin are limited to a single study which examined its effects on renal glucose and sodium handling, reporting that empagliflozin significantly increased urinary output and produced osmotic diuresis through glycosuria, with these effects occurring within first 24 hours and remaining significant throughout a 30-day period (55). Despite empagliflozin-related osmotic diuresis, an effect of empagliflozin on serum sodium levels was not observed at any time point which might be explained by the fact that the majority of participants were eunatremic at the baseline, with the lowest serum sodium on admission being 129 mmol/l (55). Conflicting data are available about the effect of dapagliflozin, another SGLT2 inhibitor, on serum sodium concentration in patients with heart failure. On the one hand, findings from a large trial suggested that dapagliflozin did not result in statistically significant increase of serum sodium in those with established hyponatremia (56). On the other hand, a small study of hospitalized patients with acute heart failure demonstrated that dapagliflozin administration for 48 hours was associated with a statistically significant mean increase in serum sodium by 3 mmol/l compared to the control group and a higher resolution rate of hyponatremia (57). In total, the effect of empagliflozin on serum sodium in patients with heart failure and established hyponatremia has not been determined yet.

## Discussion

Beyond the established role of empagliflozin as a glucose-lowering agent and the recently added indications for heart failure and CKD, the emerging question is whether empagliflozin could gain a fourth indication for the management of euvolemic hyponatremia. Recent prospective trials have provided evidence in favor of its efficacy and safety for treatment of SIAD, both in the inpatient (11) and outpatient setting (12), mediated by increased free renal water excretion as a result of glycosuria-induced osmotic diuresis. However, the small sample size and short duration of treatment limit the generalizability of these findings which warrant further exploration in large prospective trials, with several research questions remaining to be answered. Moving forward, skepticism should balance enthusiasm. Even if future studies confirm the favorable efficacy-safety profile of empagliflozin, the small magnitude of sodium increase would not render it the new wonder drug for SIAD, but rather a valuable addition to the current treatment options for SIAD. Its primary use would be as an adjunct to fluid restriction in those with mild to moderate chronic euvolemic hyponatremia, but its administration as add-on to oral urea for those with inadequate response to it may also be an effective treatment option.
